# Massive Mural Thrombus Masquerading as Myxoma

**DOI:** 10.7759/cureus.25440

**Published:** 2022-05-29

**Authors:** Masi Javeed, Hanan Gruhonjic, Dveet Patel, John Forcella, Rami Akel

**Affiliations:** 1 Internal Medicine, HCA Florida Bayonet Point Hospital, Hudson, USA; 2 Cardiology, HCA Florida Bayonet Point Hospital, Hudson, USA; 3 Interventional Cardiology, HCA Florida Bayonet Point Hospital, Hudson, USA

**Keywords:** cardio thoracic surgery, trans-esophageal echocardiogram, left atrial mass, left atrial thrombus, mural thrombus

## Abstract

A 75-year-old Caucasian female with a past medical history including insulin-dependent diabetes mellitus, hypertension, and dyslipidemia, presented to the emergency room for having palpitations for three weeks. Echocardiography revealed a very large left atrial mass mimicking myxoma. Mass was excised and examined by pathology, revealing a mural thrombus. A mural thrombus is not an uncommon mass found in the left atrium. However, it does not often present symptomatically, strongly mimics an atrial myxoma on cardiac imaging, and has rarely ever been reported to be greater than seven centimeters in any dimension. We present a case of a 75-year-old Caucasian woman with a massive, symptomatic cardiac thrombus masquerading as a myxoma on imaging.

## Introduction

Intracardiac masses present a challenging diagnostic situation. The differential for intracardiac masses is vast, including benign or malignant primary tumors, secondary metastatic tumors, vegetations, anatomic variations, implanted devices, artifacts, and thrombi [1]. The gold standard for diagnosis is a biopsy and may not always be clinically feasible [2]. As a result, non-invasive imaging modalities, including echocardiography, cardiac computed tomography, and cardiac magnetic resonance, are often useful in narrowing the differential for an intracardiac mass, though these findings are often ambiguous. We report the case of a patient with a massive ambiguous left atrial mass, suspicious of possible myxoma, later found by pathology to be a large mural thrombus.

## Case presentation

A 75-year-old Caucasian female, with a past medical history, including insulin-dependent diabetes mellitus, hypertension, and dyslipidemia, was admitted to the hospital due to three weeks of palpitations. Palpitations were intermittent and lasted about five minutes per episode. The patient denied shortness of breath, syncope, dizziness, vertigo, cough, chest pain, fevers, leg swelling or pain, nausea or vomiting, and recent stressors. The patient had been vaccinated against COVID-19. The patient endorsed minimal alcohol use but denied tobacco or other illicit drug use. The patient denied recent procedures or surgeries. The patient reported compliance with medications, including insulin, glipizide, metoprolol tartrate, and atorvastatin. 

Vitals on admission were unremarkable. On the initial physical exam, only bilateral pitting edema was notable. Initial labs, including cell blood count, comprehensive metabolic panel, and troponin, thyroid-stimulating hormone, prothrombin time, partial thromboplastin time, and international normalized ratio, were unremarkable. Chest X-ray and bilateral lower extremity venous duplex were both unremarkable. Bilateral carotid artery duplex showed only mild left carotid disease. Blood cultures were unremarkable.

Transthoracic echocardiogram showed an ejection fraction of 55-60%, grade 1 diastolic dysfunction, moderate mitral regurgitation, mild tricuspid regurgitation, mild pulmonary insufficiency, and, more notably, a large, highly mobile left atrial mass suspected to be myxoma, prolapsing into the left ventricle during diastole.

**Figure 1:**
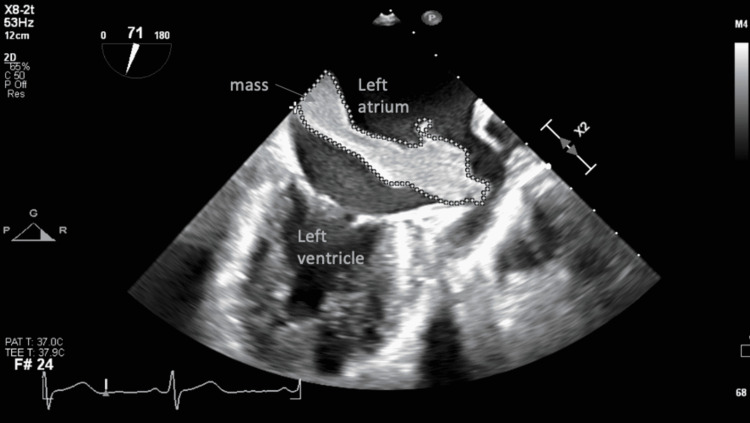
Mid-esophageal two-chamber view with omniplane angle of 71 degrees revealed a left-sided mass adjacent to the interatrial septum

**Figure 2:**
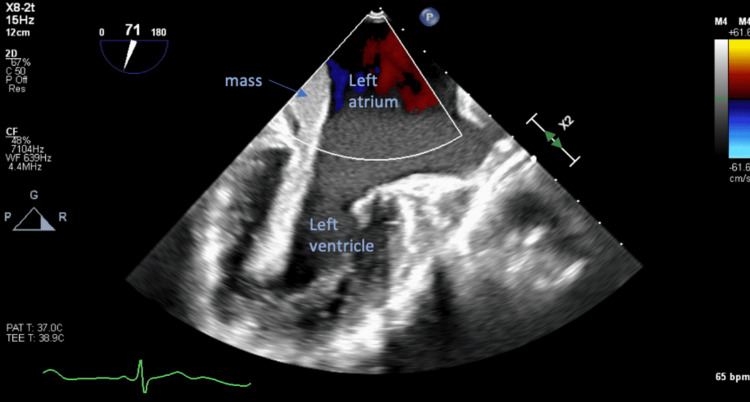
Mid-esophageal two-chamber view with omniplane angle of 71 degrees revealed the left-sided mass prolapsing into the left ventricle

Due to this mass, the patient was transferred to the ICU, and cardiothoracic surgery was consulted. Upon further investigation, a transesophageal echocardiogram showed only mild regurgitation and again a definite, large, irregular, highly mobile mass on the left side of the interatrial septum, which was likely to be a myxoma (Figures 1-2).

**Figure 3:**
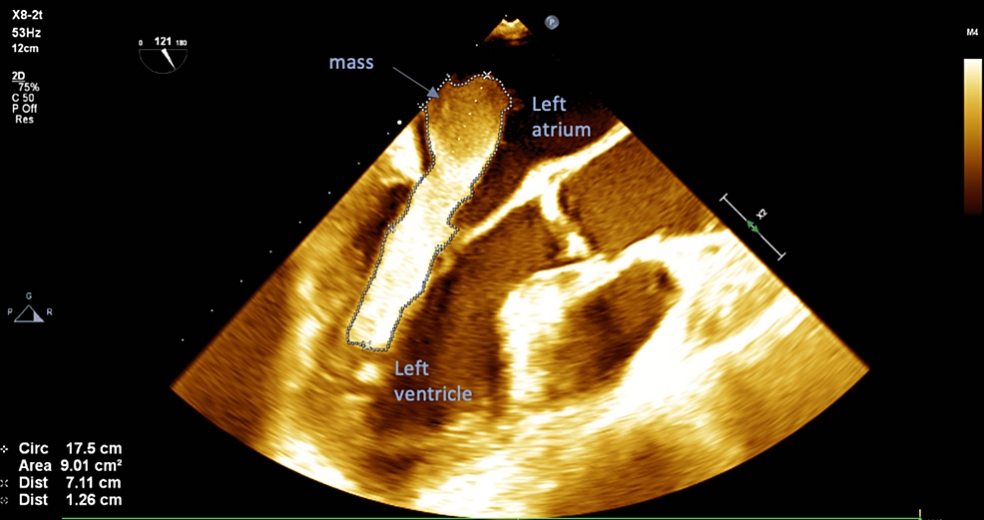
Mid-esophageal aortic valve long-axis view revealed the left-sided mass to be 9.01 square centimeters in area and 7.11 centimeters in length

**Figure 4:**
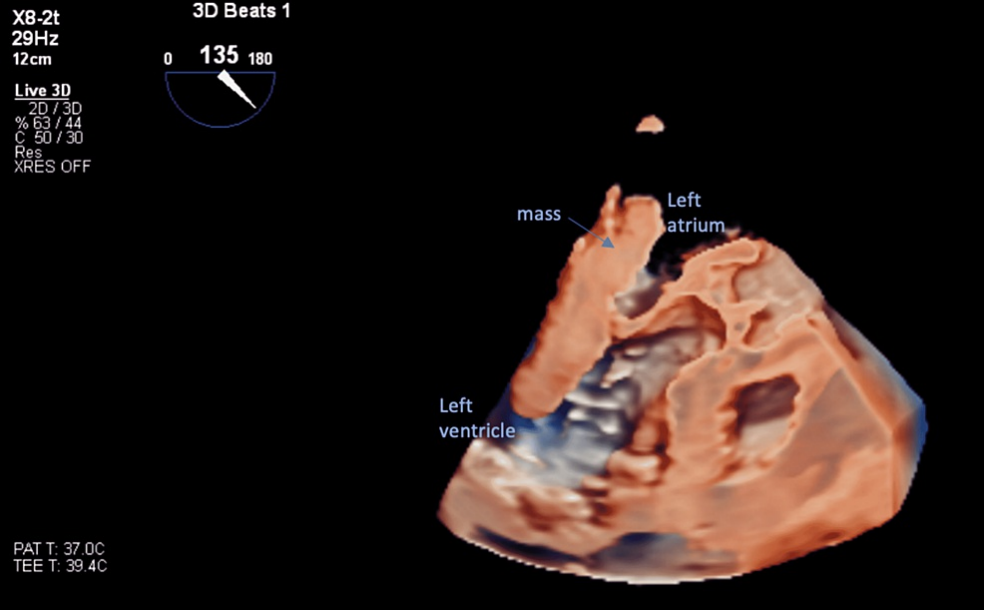
Three-dimensional transesophageal echocardiogram with mid-esophageal aortic valve long-axis view revealed the left atrial mass prolapsing into the left ventricle

We had a discussion with the patient and her family at this point in regards to possible surgery, and consent was taken from them. Cardiac catheterization was performed and showed minor luminal irregularities in the left main coronary artery, 20% proximal disease of the left anterior descending artery, 20% disease of the first obtuse marginal artery, 30-40% lesion of the proximal right coronary artery, and 60% lesion of the mid-right coronary artery. As such, no percutaneous coronary intervention was required, and excision of the mass was subsequently performed. A very large, greater than 7-centimeter gelatinous mass occupying most of the left atrium and protruding through the mitral valve into the left ventricle was removed; it was found to be attached with a wide base to the septum inferiorly away from the mitral valve on peri-operative transesophageal echocardiogram (Figures 3-4).

Two chest tubes were placed in the mediastinum and the right pleural space, as well as one temporary right ventricle pacing wire. Pathology revealed the specimen predominately consisted of fibrin with only mild increased vascularity indicating a mural thrombus; features of a mixed cell tumor were not identified.

With the exception of postoperative atrial fibrillation, which converted to normal sinus rhythm after a bolus of intravenous amiodarone and metoprolol tartrate, the patient did well postoperatively and was soon discharged home on oral apixaban. 

## Discussion

Thrombosis refers to the partial or complete blockage of a blood vessel secondary to the clotting of blood. Intracardiac thrombi, like all thrombotic events, are resultant of a triad of factors that lead to the clotting of blood: stasis, activation of the coagulation cascade, and endothelial damage. These thrombi are clinically significant due to their arrhythmogenicity and inherent risk of systemic embolization with resulting stroke, renal infarction, splenic infarction, or mesenteric infarction. It is consequently crucial to successfully differentiate intracardiac thrombus from other intracardiac masses and initiate appropriate therapy. In this report, we presented the case of a massive, symptomatic left atrial thrombus measuring greater than seven centimeters, which is larger than the vast majority of similar thrombi [3-15]. 

The gold standard for diagnosis of an intracardiac mass remains surgery or biopsy [2]. In this case report, surgery followed by pathological examination revealed the left atrial mass to be a large thrombus. However, this is not always feasible, and non-invasive imaging is often used as a surrogate. The differential diagnosis for intracardiac mass is broad and often includes primary cardiac tumors, such as atrial myxoma and intracardiac thrombus [1]. There are several radiologic features used to differentiate the two entities. On echocardiography, atrial myxomas typically present as a mobile atrial mass with a distinct narrow stalk usually anchored to the fossa ovalis. Intracardiac thrombi are alternatively usually found on the left atrial appendage, are usually smaller in size, and exhibit less mobility [16].

Despite the final diagnosis, the mass in this case report exhibited several features concerning cardiac tumor, especially confounding the clinical picture. Though there are multiple intracardiac thrombi documented in the literature, there are very few documented over six centimeters in any given dimension [3-15]. An intracardiac thrombus seven centimeters in one dimension is especially rare, as larger sizes usually portend a cardiac tumor [16]. In addition, the thrombus in our case was located near the interatrial septum, which is unique as most thrombi originate in the atrial appendage. Also, the thrombus exhibited high mobility, a feature usually found in myxomas [16]. 

Despite the findings in this case report, echocardiography has reportedly high accuracy in differentiating cardiac masses [17,18]. Additionally, cardiac computed tomography and magnetic resonance have been shown to be useful adjunctive tools [19]. However, while these imaging modalities can be helpful in guiding management, they are not adequate replacements for surgery or biopsy and histologic analysis in especially complex cases. 

## Conclusions

In this case, we found a massive left atrial thrombus masquerading as a myxoma due to a variety of characteristics on echocardiography. This highlights the difficulty in differentiating cardiac masses only through imaging and without surgery or biopsy and pathology. In an attempt to avoid surgery, one proposed approach for the treatment of intracardiac thrombi is treatment with systemic anticoagulation and repeat imaging. Further investigations could include retrospective or longitudinal studies assessing outcomes in these cases.

## References

[1] Aggeli C, Dimitroglou Y, Raftopoulos L (2020). Cardiac masses: the role of cardiovascular imaging in the differential diagnosis. Diagnostics.

[2] Bussani R, Castrichini M, Restivo L (2020). Cardiac tumors: diagnosis, prognosis, and treatment. Curr Cardiol Rep.

[3] Jang KH, Shin DH, Lee C, Jang JK, Cheong S, Yoo SY (2010). Left atrial mass with stalk: thrombus or myxoma?. J Cardiovasc Ultrasound.

[4] Tasar M, Ada F (2020). Left atrial mass: Thrombus? Myxoma? or Both? Myxoma surrounded by thrombus. Cardiol Vasc Res.

[5] Lu H, Nordin R, Othman N (2016). Biatrial thrombi resembling myxoma regressed after prolonged anticoagulation in a patient with mitral stenosis: a case report. J Med Case Rep.

[6] Strecker T, Rümmele P, Seitz T, Nooh E (2017). A huge left atrial mass "not a myxoma". Cardiol J.

[7] Khan N, Whitton A, Hoffman R, Qualtieri J, Dunlap S (2014). Left atrial thrombus mimicking atrial myxoma on imaging studies in a patient with cardiac transplant. J Cardiol Cases.

[8] Samson A, Taquiso J, Uy C (2018). Right atrial mass mimicking a myxoma in a patient with systemic lupus erythematosus. J Am Coll Cardiol.

[9] Dhawan S, Tak T (2004). Left atrial mass: thrombus mimicking myxoma. Echocardiography.

[10] Morelli S, Testa G, Voci P (1996). Large left atrial thrombus mimicking atrial myxoma: successful treatment with anticoagulant therapy. Eur Heart J.

[11] Adauy JV, Gabrielli L, Córdova S, Saavedra R, McNab P (2017). Big thrombus "sitting" in an atrial septal aneurysm. Echocardiography.

[12] Shimamoto K, Kawagoe T, Dai K, Inoue I (2014). Thrombus in the left atrial septal pouch mimicking myxoma. J Clin Ultrasound.

[13] Peters F, Khandheria BK, Patel AR, Essop MR (2013). Mitral stenosis and pedunculated left atrial thrombus: an unusual presentation. Eur Heart J Cardiovasc Imaging.

[14] Kodali S, Yamrozik J, Biederman RW (2010). Left atrial thrombus masquerading as a myxoma in a patient with mitral stenosis. Echocardiography.

[15] Chen MA, Ahlgrim AA, Allen CT (2009). Location, location, location: a left atrial mass. J Am Soc Echocardiogr.

[16] Nemani L, Killi S (2017). Right atrial thrombus mimicking myxoma. Indian J Cardiovasc Dis Women.

[17] Ragland MM, Tak T (2006). The role of echocardiography in diagnosing space-occupying lesions of the heart. Clin Med Res.

[18] Vincelj J, Sutlic Z, Biocina B, Nikić N, Lajtman Z (2001). Diagnostic accuracy of transesophageal echocardiography for detection of atrial masses. Acta Medica Croatica.

[19] Scheffel H, Baumueller S, Stolzmann P, Leschka S, Plass A, Alkadhi H, Schertler T (2009). Atrial myxomas and thrombi: comparison of imaging features on CT. AJR Am J Roentgenol.

